# The cost of healthier and more sustainable food choices: Do plant-based consumers spend more on food?

**DOI:** 10.1186/s40100-022-00224-9

**Published:** 2022-07-26

**Authors:** Daniel Francisco Pais, António Cardoso Marques, José Alberto Fuinhas

**Affiliations:** 1 Management and Economics Department, NECE-UBI, University of Beira Interior, Rua Marquês d’Ávila e Bolama, 6201-001 Covilhã, Portugal; 2 NECE-UBI, CeBER and Faculty of Economics, University of Coimbra, Av. Dr. Dias da Silva 165, 3004-512 Coimbra, Portugal

**Keywords:** Food economics, Consumer behaviour, Food expenditure, Food choices, Plant-based consumers, Survey-based analysis

## Abstract

Plant-based diets are often promoted as healthier and more sustainable and thus as a mechanism to achieve the targets proposed to mitigate climate change and noncommunicable diseases. However, plant-based diets can be perceived as more expensive than the common omnivorous diets, when considering the expensive novel meat substitutes and also the higher costs of fruits and vegetables, whose consumption is perceived to increase. Therefore, the present study assesses the question: Do plant-based consumers spend more on food compared to omnivorous consumers? Based on primary data (*n* = 1040) collected through an online survey, representative of the Portuguese population, through logistic regressions, it was possible to conclude that plant-based consumers, particularly vegan, are associated with lower food expenditures compared to omnivorous consumers. In fact, plant-based consumers are shown to spend less than all other consumers assessed. Food policies aligning healthiness and sustainability with affordability can deliver a major boost for the promotion of plant-based diets and help achieve the mitigation targets proposed.

## Introduction

Current demand for food is putting pressure on the environment, threatening present and future generations. According to the Food and Agricultural Organisation (FAO) data, animal-based consumption is growing worldwide, particularly in population-dense middle-income countries (FAO 2022). This global excessive consumption could have significant impacts on the environment and public health, directly through consumption and indirectly through climate change (Godfray et al. 2018). Following the latest research, the food sector alone accounts for one-third of global anthropogenic greenhouse gas emissions (Crippa et al. 2021). To achieve the targets proposed to mitigate climate change, many international organizations have highlighted the need for a shift in dietary habits. The Intergovernmental Panel on Climate Change (IPCC) highlights that diets with larger shares of animal-based foods have lower mitigation potentials, compared with plant-based diets (Mbow et al. 2019). The literature also points out that animal-based foods, in general, have higher ecological footprints compared with plant-based diets (Clune et al. 2017; Poore and Nemecek 2018; Reinhardt et al. 2020).

Plant-based diets are addressed in the literature as both healthier and more sustainable (Chen et al. 2019; Clark et al. 2019; Springmann 2019), with the Academy of Nutrition and Dietetics defending that, if planned and diversified, they can satisfy all nutritional requirements (Melina et al. 2016). Furthermore, the World Health Organization (WHO) has also classified processed meat as “carcinogenic to humans” and red meat as “probably carcinogenic”, recommending a reduction in these types of meat followed by an increase in plant-based foods (Bouvard et al. 2015). More recently, two new studies revealed that a healthy plant-based diet rich in whole foods could help protect against the COVID-19, suggesting that consumers following the diet are both less likely to catch the virus and be hospitalized with it (Kim et al. 2021; Merino et al. 2021). This relationship has been recently highlighted by Kahleova and Barnard (2022).

Similar to the IPCC, the Lancet Commission, in their extensive review on food consumption, has advised for a drastic reduction in animal-based consumption, particularly red meat, and a twofold increase in plant-based foods, particularly fruits and vegetables (Willett et al. 2019). The European Union Farm to Fork Strategy notes that “moving to a more plant-based diet with less red and processed meat and with more fruits and vegetables will reduce not only risks of life threatening diseases, but also the environmental impact of the food system” (European Commission 2020). Additionally, Vanham et al. (2018) and Gibin et al. (2022) suggest that this shift also substantially reduces consumption of water resources.

Berners-Lee et al. (2018) further advise that radical changes to the dietary choices of most consumers are required so that current global food production is sufficient to meet the nutritional needs of the projected global population of 9.7 billion in 2050. For particular population groups, such as pregnant women and children, the scientific literature suggests that a well-planned plant-based diet, using supplementation when needed, may be considered safe (Baroni et al. 2019; Sebastiani et al. 2019; Sutter and Bender 2021). These recommendations are also in line with the ones from the Academy of Nutrition and Dietetics above-mentioned. Regarding the population of athletes, the scientific literature also suggests that a nutritive vegan diet can be designed to achieve the dietary needs of most of this population satisfactorily (Rogerson 2017; Shaw et al. 2022).

Considering the negative externalities associated with the excessive animal-based consumption, a dietary shift away from these ecologically burdensome foods towards greater reliance on more sustainable and healthier ones, particularly healthy plant-based foods, is required (Aiking and de Boer 2020; Jiang et al. 2020). Even for animal-based foods produced following sustainable methods, plant-based foods still show lower ecological footprints. Poore and Nemecek (2018) reveal that the least ecologically burdensome red meats, in terms of greenhouse gas emissions, are still higher than any plant-based food compared for the same 100 g intake of protein. However, concerning plant-based foods, nuts in particular are still dominated by low-yielding cashews and water-, fertilizer- and pesticide-intensive almonds. Rice production is also a strong contributor to water footprint in Eastern countries where consumption is also high (Fan et al. 2022).

Nevertheless, Rabès et al. (2020) highlight that omnivorous consumers have the highest environmental impact, and vegans the lowest, and further that an organic-based omnivorous diet has a higher environmental impact than a conventional one (see also Chai et al. (2019)). Additionally, Mottet et al. (2017) highlight that animals have relatively low efficiency in converting feed into human-edible foods, more so when considering that feed rations may contain human-edible food and that these feed rations are competing for land suitable for human-edible food production. Particularly, the authors estimate that to produce 1 kg of boneless requires an average of 2.8 kg and 3.2 kg of human-edible feed in ruminant and monogastric systems, respectively. The authors conclude that farming practices in the livestock sector do not account much for the environmental impact of animal-based foods, and that plant-based foods, and the correspondent plant-based diets, score the lowest environmental impacts. Although the impact of animal-based foods can be mitigated mainly by changes in behaviour, the impact of plant-based foods can be further lowered through technology change and innovation in efficiency rates.

To promote the dietary transition proposed, it is vital to understand the motivators behind food choices. Knowing the reasons behind food choices, and particularly for abstaining meat or choosing more plant-based foods, which vary across consumers and may involve taste, health, animal welfare, the environment, among other considerations, is crucial to define effective policy measures (Neff et al. 2018; Graça et al. 2019; Dominici et al. 2021; Martinelli and De Canio 2021; Hielkema and Lund 2021). From the various motivators, regularly the issue of cost arises as price is often a driver of the dietary transition (Pais et al. 2021). From the many plant-based consumers, an “economic vegetarian” is one who avoids meat because it is more expensive (Lusk and Norwood 2016). On the retailers’ side, Tjärnemo and Södahl (2015) suggest that food retailers are not open to guide consumers to more environmental-friendly food choices as it would mean reducing meat consumption (see also Bălan (2021)). Food retailers further suggest that animal-based foods are important to attract new and keep loyal customers.

With the recent meat-substitute market reaching about 12% and increasing rapidly (Martinelli and De Canio 2021), reaching $7.5bn by 2025 (Allied 2019), the public might generally perceive plant-based foods as more expensive than their animal counterparts, and consequently perceiving plant-based diets as more expensive. Additionally, fruits and vegetables may also be out of reach for some consumers due to high prices (Li et al. 2018). However, studies such as Stewart et al. (2021) report that if consumers prioritize fruits and vegetables in their budgets, they can better afford to meet the dietary lines regarding the consumption of these foods. Nevertheless, Grabs (2015) and Berners-Lee et al. (2012) estimate that plant-based consumers save up to 10% and 14% on grocery store expenditure compared to omnivorous. However, these estimations are based on a pooled data set and assumptions using inferred prices, rather than observed prices paid by consumers. This method does not assess the specific meals consumed from different diets, which is a major gap in the literature.

To the best of the authors’ knowledge, only one published peer-reviewed study has assessed food expenditures at the consumer level for different diets. Using primary data for the USA, Lusk and Norwood (2016) conclude that consumers who follow a strictly plant-based diet (vegan) report lower food expenditures, and that plant-based diets are in practice cheaper than the omnivorous diets. However, consumer preferences and the food market may differ considerably between the USA and Europe, and particularly for Mediterranean countries such as Portugal where the Mediterranean diet is greatly promoted. Thus, the omnivorous diet may show considerable differences between these regions. No evidence has been assessed at the consumer level in Mediterranean countries, which presents an opportunity for the present study, more so when the Portuguese food guidelines suggest that the share of animal-based foods, such as meat, fish and eggs, should not exceed 5% (DGS and FCNAUP 2017), and the current average consumption exceeds 15% (FAO 2022). As a result, food consumption is the main driver (30%) of Portugal’s ecological footprint (Galli et al. 2020b). Portugal requires 2.3 planets Earth to satisfy a year of consumption (Galli et al. 2020a).

Considering the gap in the knowledge highlighted, more assessments are needed to fully understand the implications of the shift towards less animal-based foods and more plant-based diets globally, proposed by international organizations and the scientific literature. The implications of the potential dietary costs of plant-based diets go beyond its effect on a consumer’s finances—it will also be crucial for the assessments of the ecological footprint of the proposed shift. As described by Grabs (2015), “the rebound effects” of changing diets shows that one half of the carbon footprint reduction credited to the shift towards plant-based diets actually disappears after considering the re-spending of the savings (see also Lekve Bjelle et al. (2018)). If plant-based diets have both a lower ecological footprint and a lower price tag, it is crucial to account for the possibility such re-spending. Thus, a clear understanding of the relationship between plant-based diets and food expenditures is vital as it helps to recognize the economic consequences of shifting diets, as well as its potential environmental consequences. Additionally, if plant-based diets are cheaper than other diets, they could be a potential mechanism to mitigate food insecurity.

Following the above, the present study aims to verify if plant-based consumers in fact spend more on food than omnivorous consumers, at the consumer level, in the context of a Mediterranean country. In this paper, both food expenditures at-home and away-from-home are assessed. Besides diets, other consumers’ characteristics are tested such as preference for organic foods, making use of leftovers, having own production, and also socio-economic characteristics such as age, education level, household disposable income, among others.

The study is structured as follows: besides the motivation in Sect. 1, Sect. 2 displays the data and methods applied. Then, results are presented and further discussed in Sect. 3. Finally, Sect. 4 concludes and examines drawbacks and future research.

## Data and methods

### Respondents and sample procedure

This research uses primary data collected through a cross-sectional online survey,^1^ which was constructed based on the recent literature on food choices, particularly the Meat Demand Monitor^2^ (Tonsor et al. 2021). From February to April 2021, a total of 2332 completed responses were collected, of which 204 were discarded due to inconsistencies in the answers throughout the survey. From the remaining 2128, 126 participants responded not knowing how much they spend on food per week, while another 113 reported that they are not the ones who do the shopping (potentially giving biased estimates of food expenditure). Considering that the goal of the analysis is focused on food expenditure, these responses (mostly from teenagers) were also discarded. A total sample size of 1889 respondents was used. Subsequently, to guarantee a representative sample of consumers in Portugal, quota sampling was performed considering gender, age, region, and education. Taking advantage of an algorithm that optimized the representativeness of the sample, considering the demographics mentioned, a representative subsample of active Portuguese consumers was built with a total of 1040 responses.

The size of the subsample was achieved considering the population size, approximately 6.619.000 consumers between the age of 15 and 64 in 2019 according to census estimated data, and a sampling error of 4% with a 99% confidence interval, which was calculated through the following formula:

$${\text{Sample size}} = \frac{{\frac{{z^{2} *p\left( {1 - p} \right)}}{{e^{2} }}}}{{1 + \left( {\frac{{e^{2} *p\left( {1 - p} \right)}}{{e^{2} *N}}} \right)}},$$

where *z* denotes the *z*-score (the number of standard deviations a given proportion is away from the mean related to the desired confidence interval), $e$ denotes the margin of error, and *N* the population size to be assessed.

Following Table 1, the subsample is broadly representative of the Portuguese population by gender, age, and region, with a slightly more educated demographic common in online and overall surveys as the literature shows (Driediger and Bhatiasevi 2019; Hood et al. 2020; Cao et al. 2021; Hielkema and Lund 2021).

**Table 1:** Demographic characteristics of survey responses

	Sample (1889) (%)	Subsample (1040)	Portugal* (%)	∆**
*Gender*				
Male	24.25	42.79% (445)	48.25	− 5.46
Female	75.60	56.92% (592)	51.75	5.17
Non-binary	0.15	0.29% (3)	–	–
*Age*				
15–19	5.98	8.46% (88)	8.24	0.22
20–24	18.00	12.60% (131)	8.32	4.28
25–29	13.29	8.27% (86)	8.27	0.00
30–34	10.27	8.56% (89)	8.56	0.00
35–39	9.95	10.19% (106)	10.16	0.03
40–44	11.38	11.83% (123)	11.85	− 0.02
45–49	11.17	10.96% (114)	11.93	− 0.97
50–54	8.68	11.25% (117)	11.26	− 0.01
55–59	7.57	11.15% (116)	11.18	− 0.03
60–64	3.71	6.73% (70)	10.23	− 3.50
*Regions*				
Norte	40.18	35.87% (373)	35.88	− 0.01
Centro	21.28	21.25% (221)	21.25	0.00
Alentejo	6.99	6.63% (69)	6.61	0.02
Lisboa	20.21	26.83% (279)	26.84	− 0.01
Algarve	4.98	4.13% (43)	4.18	− 0.05
Açores	2.38	2.6% (27)	2.56	0.04
Madeira	4.08	2.69% (28)	2.69	0.00
*Education*				
Higher education	72.84	59.42% (618)	25.38	34.04

*2019 census estimated data from the Statistics National Institution (www.ine.pt)

**Difference between subsample and population

### Variables, model specification, and hypotheses

Two variables are used to assess food expenditure: food expenditure at-home (*FEAH*) and food expenditure away-from-home (*FEAFH*). The participants were asked “On average, what is your weekly expenditure on food to consume at home?” and a similar question was asked for food away-from-home. These variables were recorded as ordinal with eight categories each, as described in Table 2.

**Table 2:** Summary of food expenditure ordinal variables

#	*FEAH*	Responses (Obs.)	*FEAFH*	Responses (Obs.)
1	Less than 20€	3.08% (32)	Less than 5€	25.38% (264)
2	20€–39€	10.77% (112)	5€–9€	17.31% (180)
3	40€–59€	20.19% (210)	10€–19€	20.77% (216)
4	60€–79€	14.81% (154)	20€–39€	18.85% (196)
5	80€–99€	16.35% (170)	40€–59€	8.56% (89)
6	100€–119€	14.90% (155)	60€–79€	4.33% (45)
7	120€–139€	7.60% (79)	80€–99€	2.21% (23)
8	More than 140€	12.31% (128)	More than 100€	2.60% (27)

To achieve the goal of understanding if plant-based diets are more expensive than other diets, such as the omnivorous one, a nominal variable for consumers’ current diet (*DIET*) is assessed. The variable distinguishes between five categories, namely omnivorous (consumes every type of foods, animal-based and plant-based), pescatarian (fish-based, excludes meat), flexitarian (mainly plant-based, and significant reduction in animal-based foods), ovo-lacto-vegetarian (plant-based, includes animal-based by-products), and vegan (plant-based, excludes all types of animal-based foods). To further analyse the cost of different food choices, five additional variables are assessed concerning the frequency of meals consumed per week^3^ containing red meat (MRED), white meat (MWHT), fish (FISH), ovo-lacto-vegetarian foods (OLVG), and vegan foods (VEGA). Additionally, a group of covariates, i.e., variables that affect the response variable, but which are not the primary focus of the study, are also assessed. A summary of all variables is revealed in Table 3.

**Table 3:** Summary of the variables used

Variable	Description	Type	Mean	SD	Min	Max
Dependent variables						
*FEAH*	Food expenditure at-home	O	4.672	1.968	1	8
*FEAFH*	Food expenditure away-from-home	O	3.027	1.765	1	8
Food choices						
*DIET*	Current diet	N	1.45	0.982	1	5
*MRED*	Red meat meals	O^c^	2.498	0.977	1	5
*MWHT*	White meat meals	O^c^	3.165	1.159	1	5
*FISH*	Fish meals	O^c^	2.861	1.004	1	5
*OLVG*	Ovo-lacto-vegetarian meals	O^c^	3.311	1.127	1	5
*VEGA*	Vegan meals	O^c^	2.752	1.394	1	5
Covariates						
*AGE*	Age	C	39.437	13.796	15	64
*BMI*	Body mass index	C	24.913	4.492	13.84	44.19
*K12*	1 if kids under 12	B	0.244	0.430	0	1
*EDU*	1 if higher education	B	0.592	0.492	0	1
*SGL*	1 if single	B	0.466	0.499	0	1
*FAM*	1 if family	B	0.839	0.367	0	1
*STD*	1 if student	B	0.254	0.435	0	1
*HRS*	Working hours	C	31.798	16.745	0	90
*SHOP*	1 if goes shopping for the household	B	1.834	0.373	0	1
*COOK*	1 if cooks	B	0.915	0.278	0	1
*INFO*	1 if looks for info	B	0.716	0.451	0	1
*BIO*	1 if favours bio	B	0.696	0.460	0	1
*LFTO*	1 if uses leftovers	B	0.578	0.494	0	1
*OFP*	1 if owns food production	B	0.399	0.490	0	1
*FAFH*	Meals away-from-home consumed	C	2.070	2.175	0	10
*LBOX*	Meals AFH in lunchbox consumed	C	2.116	2.975	0	10
*FRTE*	Meals ready-to-eat consumed	C	1.106	1.699	0	10
*PRC*	Consumer importance given to price	O^c^	4.057	0.845	1	5
*AWAR*	Food awareness construct^b^	O^c^	3.687	0.650	1.07	5
*EXPB*	Expenditure on plant-based (%)	O^c^	2.103	1.047	1	5

The variables were recorded as ordinal (O), nominal (N), continuous (C), and binary (B). The binary variables’ base option is otherwise, i.e., for K12, value 1 is for if participant has kids under 12 living in the house, 0 is otherwise

^a^The variable was recorded with seven categories (< 635€; 635€–999€; 1000€–1999€; …; 6000€ <)

^b^The items used for the summated scale variable are described in Table 9, in the Appendix

^c^The ordinal variables were analysed in the models as continuous

In order to assess the relationship between food choices and the expenditure on food (at-home and away-from-home), two logistic models are estimated for each food expenditure variable. One of them analyses the effect of current diets, and the other the frequency of meals, by type of food. The general model can be expressed as

1 $$y_{i} = \alpha_{i} + \beta x_{i} + \delta z_{i} + \varepsilon_{i} ,$$

where *y*_*i*_ is the ordinal dependent variable for respondent *i*. The main variables of interest concerning food choices are denoted by *x*_*i*_, where *β* is their respective parameter to be estimated; *z*_*i*_ is a vector of the covariates described in Table 3 with the respective *δ* parameter; *α* is the intercept; and *ε* is the error term. According to both AIC and BIC penalized likelihood criteria, the logit model is preferred when compared with the probit model.^4^ Thus, considering the ordinal dependent variable and both AIC and BIC criteria, the ordered logit model was used. The general specification of each model is as follows:

2 $$\begin{aligned} FEAH_{i} & = \alpha_{i} + \mathop \sum \limits_{j = 1}^{5} \mathop \sum \limits_{k = 1}^{5} \beta_{1jk} DIET_{i} + \delta_{1} AGE_{i} + \delta_{2} BMI_{i} + \delta_{3} K12_{i} + \delta_{4} EDU_{i} + \delta_{5} SGL_{i} \\ & \quad + \delta_{6} FAM_{i} + \delta_{7} STD_{i} + \delta_{8} INC_{i} + \delta_{9} HRS_{i} + \delta_{10} SHOP_{i} + \delta_{11} COOK_{i} \\ & \quad + \delta_{12} INFO_{i} + \delta_{13} BIO_{i} + \delta_{14} LFTO_{i} + \delta_{15} OFP_{i} + \delta_{16} FAFH_{i} \\ & \quad + \delta_{17} LBOX_{i} + \delta_{18} FRTE_{i} + \delta_{119} PRC_{i} + \delta_{20} AWAR_{i} + \delta_{21} EXPB_{i} + \varepsilon_{i} , \\ \end{aligned}$$

where subscript *j* denotes the base outcome and *k* the analysed outcome.

3 $$FEAFH_{i} = f\left( {DIET_{i} ; covariates} \right),$$

4 $$\begin{aligned} FEAH_{i} & = \alpha_{i} + \beta_{1} MRED_{i} + \beta_{2} MWHT_{i} + \beta_{3} FISH_{i} + \beta_{4} OLVG_{i} + \beta_{5} VEGA_{i} + \delta_{1} AGE_{i} \\ & \quad + \delta_{2} BMI_{i} + \delta_{3} K12_{i} + \delta_{4} EDU_{i} + \delta_{5} SGL_{i} + \delta_{6} FAM_{i} + \delta_{7} STD_{i} \\ & \quad + \delta_{8} INC_{i} + \delta_{9} HRS_{i} + \delta_{10} SHOP_{i} + \delta_{11} COOK_{i} + \delta_{12} INFO_{i} + \delta_{13} BIO_{i} \\ & \quad + \delta_{14} LFTO_{i} + \delta_{15} OFP_{i} + \delta_{16} FAFH_{i} + \delta_{17} LBOX_{i} + \delta_{18} FRTE_{i} \\ & \quad + \delta_{119} PRC_{i} + \delta_{20} AWAR_{i} + \delta_{21} EXPB_{i} + \varepsilon_{i} , \\ \end{aligned}$$

5 $$FEAFH_{i} = f\left( {MRED_{i} ;MWHT_{i} ;FISH_{i} ;OLVG_{i} ;VEGA_{i} ;covariates} \right).$$

Following the objectives stated earlier, the empirical hypotheses to be tested are formalized as follows:

#### **H1**

Following a plant-based diet, compared to omnivorous diets, positively affects the likelihood of spending more on food:

- Plant-based consumers spend more on food than omnivorous consumers in terms of food expenditures at-home (H1a: Eq. 2, $\beta_{115} > 0)$
- Plant-based consumers spend more on food than omnivorous consumers in terms of food expenditures away-from-home (H1b: Eq. 3, $\beta_{115} > 0)$

#### **H2**

Higher frequency of plant-based meals positively affects the likelihood of spending more on food:

- Eating more plant-based meals is associated with a higher food expenditure at-home (H2a: Eq. 4, $\beta_{5} > 0$)
- Eating more plant-based meals is associated with a higher food expenditure away-from-home (H2b: Eq. 5, $\beta_{5} > 0$).

In addition to the two main hypotheses concerning diets, three other hypotheses are tested, particularly:

#### **H3**

Informed consumers frequently spend less on food at-home (H4a: Eq. 4, $\delta_{12} < 0$) and away-from-home (H4b: Eq. 5, $\delta_{12} < 0$).

#### **H4**

Consumers who favour biologic/organic foods tend to spend more on food at-home (H5a: Eq. 4, $\delta_{13} > 0$) and away-from-home (H5b: Eq. 5, $\delta_{13} > 0$).

#### **H5**

Consumers who cook, use leftovers, and own/receive local food production frequently spend less on food at-home (H6a: Eq. 4, $\delta_{11} < 0 \wedge \delta_{14} < 0 \wedge \delta_{15} < 0$) and away-from-home (H6b: Eq. 5, $\delta_{11} < 0 \wedge \delta_{14} < 0 \wedge \delta_{15} < 0$).

Hypotheses *H1* and *H2* make use of Eqs. 1, 2 and 3, 4, respectively, while the rest of the hypotheses make use of Eqs. 4–5 since the covariates’ effects are highly similar between Eqs. 2, 3 and 4, 5. After estimating the coefficients, the marginal effects are computed as follows:

$$\frac{{\partial \hat{D}_{i} }}{{\partial X_{ji} }} = \hat{\beta }_{j} \hat{D}_{i} \left( {1 - \hat{D}_{i} } \right).$$

The STATA command used to compute the marginal effects is *mchange*. (For more details, see Long and Freese (2014).) Both coefficients and marginal effects are analysed in the next section.

## Results and discussion

### Current diets and food expenditure

Current diets were directly addressed in the survey through the question “Which of the following diets do you identify with?”, with the five diets mentioned earlier and an additional “other” option as possible answers. Moreover, using the answers regarding meals per week for each primary food, it was possible to identify the current diets with greater robustness. A comparison of results between both questions showed some dissonance, where the self-identified (perceived) diet was different from the actual diet (considering the meals consumed per week). Table 4 shows the differences.

**Table 4:** Comparison between *perceived diet* and *actual diet*

*Perceived diet*	*Actual diet*					
	Omnivorous	Pescatarian	Flexitarian	OLVeg	Vegan	Total
Omnivorous	832	0	0	0	0	832
Pescatarian	0	5	22	0	0	27
Flexitarian	0	19	76	0	0	95
O-L-Vegetarian	0	9	11	26	0	46
Vegan	0	1	5	8	26	40
Total	832 (80%)	34 (3.3%)	114 (10.9%)	34 (3.3%)	26 (2.5%)	1040

Some self-identified plant-based consumers reported eating animal-based foods at least once per week. Of the 40 self-identified vegans, in practice, 8 are ovo-lacto-vegetarians, 5 are flexitarians, and 1 is pescatarian, reducing the number of actual vegans to 26. The number of vegetarians also decreases, while flexitarians and pescatarians increase in number. It is not uncommon for participants to report being plant-based and also report sporadically consuming animal-based foods, as observed in the seminal work by Dietz et al. (1995). In fact, past studies have also identified this dissonance. Lusk and Norwood (2016) report that 5.2% are vegetarian, but only 2.2% are actual plant-based consumers. Self-identified plant-based consumers may tend to eat less animal-based foods. However, they may also be more likely to pay a higher price for these foods for their higher quality (e.g., organic, ethical). Jung et al. (2022) suggest that when the price of cultured meat was high, the purchase intention for novel food was also higher. Thus, it is vital to correct the data of actual vegetarians and vegans, as the present research does, so that this bias does not affect the results.

In the subsample assessed, 2.5% of the participants follow a vegan diet and 3.27% follow an ovo-lacto-vegetarian diet. In comparison with past studies, Centro Vegetariano and AC Nielsen (2017) reported that, in 2017, 0.6% of the population followed a vegan diet and 1.2% a vegetarian (ovo-lacto-vegetarians and vegans) diet. More recently, Jung et al. (2022) reported, for the USA, similar shares of vegans (2.2%). Thus, although the share of omnivorous is still large, 20% of the participants report eating less meat, concluding that in 4 years the numbers of alternative diets have increased considerably.

**Fig. 1:**
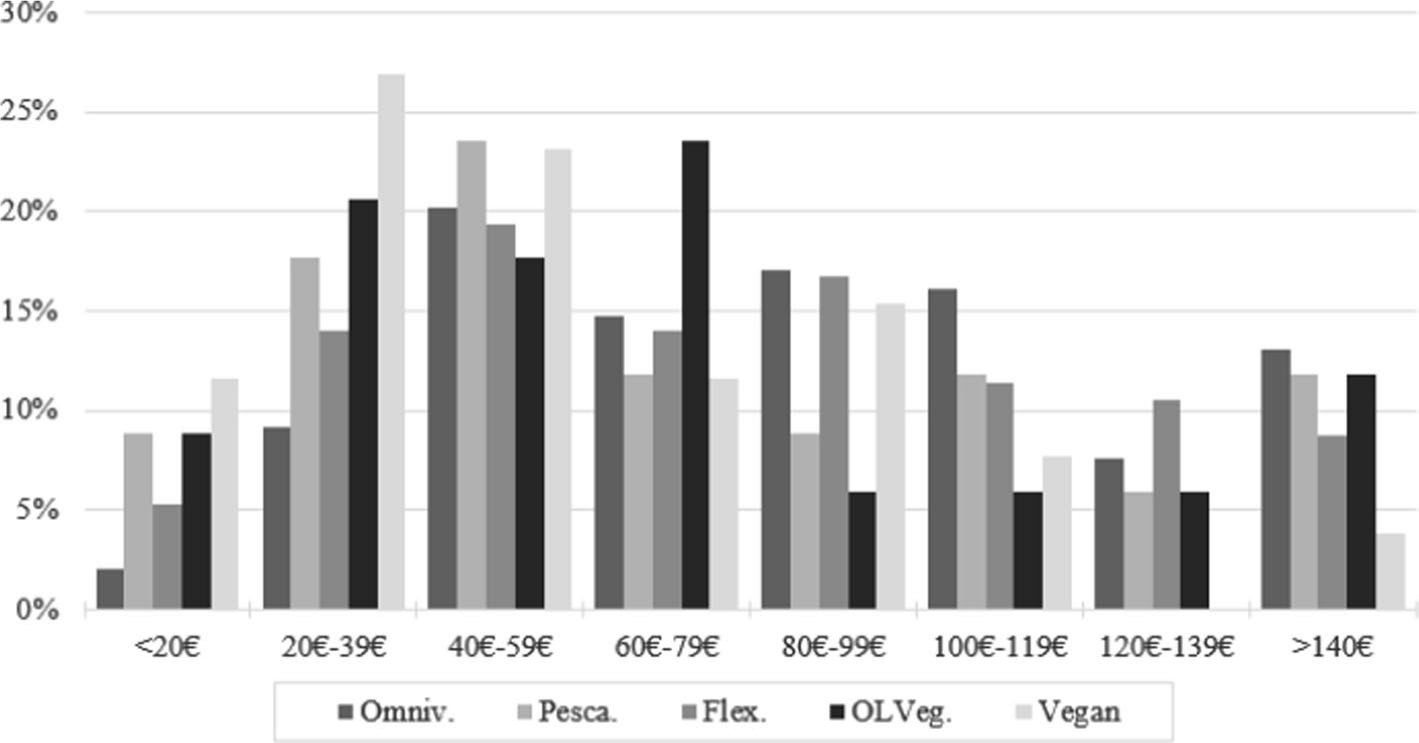
Food expenditure at-home per week by actual diets (in % of consumers of each diet)

Figure 1 depicts food expenditure at-home for all diets. In general, self-reported food expenditures are lower among vegans. For example, more than 10% of the vegans reported spending less than 20€ per week, while only 2% of omnivorous reported the same amounts. Among vegans, 23% report spending between 20€ and 39€ per week, while for pescatarians, 24% report spending between 40€ and 59€, and 24% of vegetarians reported spending between 60€ and 79€. This might be a first indication that plant-based consumers spend less on food compared to consumers following other diets. Additionally, food expenditures are generally higher for omnivorous consumers, compared to the other consumers. On average, omnivorous consumers reported a food expenditure at-home of 75.96€/week. This is the highest expenditure level compared with the other diets, particularly in comparison with 62.35€/week for pescatarians, 68.6€/week for flexitarians, 59.39€/week for ovo-lacto-vegetarians, and 47.78€/week for vegans.

**Fig. 2:**
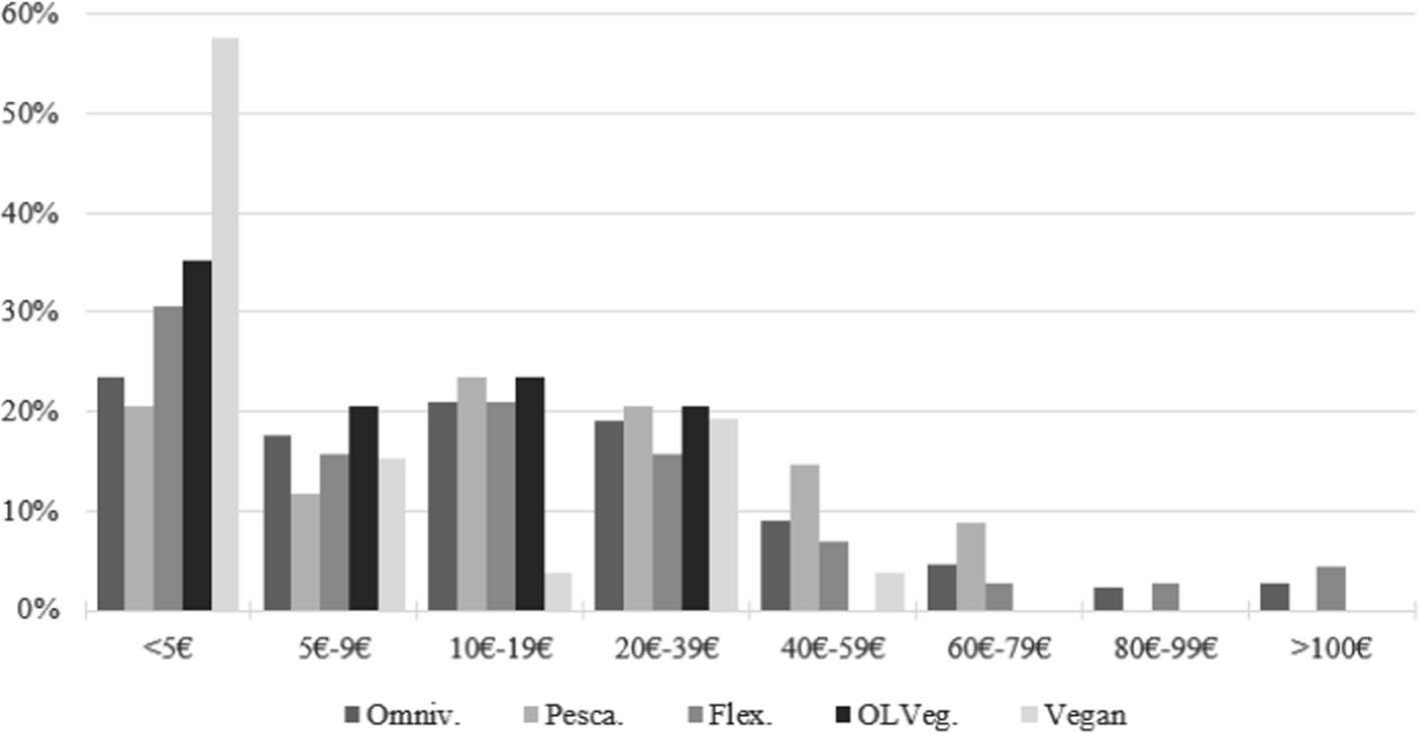
Food expenditure away-from-home among actual diets (in % of consumers of each diet)

Moreover, Fig. 2 shows the responses for the food expenditure away-from-home, which are quite different than the ones reported for expenditure at-home. Nonetheless, more than half of the vegans report spending less than 5€/week outside their home, while only 22% of animal-based consumers report spending the same amount. Considering both food expenditures at-home and away-from-home, the descriptive statistics suggest that vegan consumers may spend less on food than their omnivorous counterparts, but the same may not be concluded for ovo-lacto-vegetarians.

Dietary preferences, however, may not be the only aspect influencing food expenditures. Having children, living with family, buying food for others or not, being responsible to shop and cook, having more disposable income, are all characteristics, among others, that may also affect expenditure. Thus, demographics may play a profound role in shaping how much consumers spend on food. These aspects are also considered in the models so that the true effect of diets is estimated, minimizing the omitted-variable bias, that is, vegans may spend less because they live alone, compared to omnivorous consumers who have children. Only with the results from the empirical analysis, it is possible to ascertain which consumers in reality spend less, since the models take into account several other variables as potential confounders.

### Falsifiability of hypotheses

**Fig. 3:**
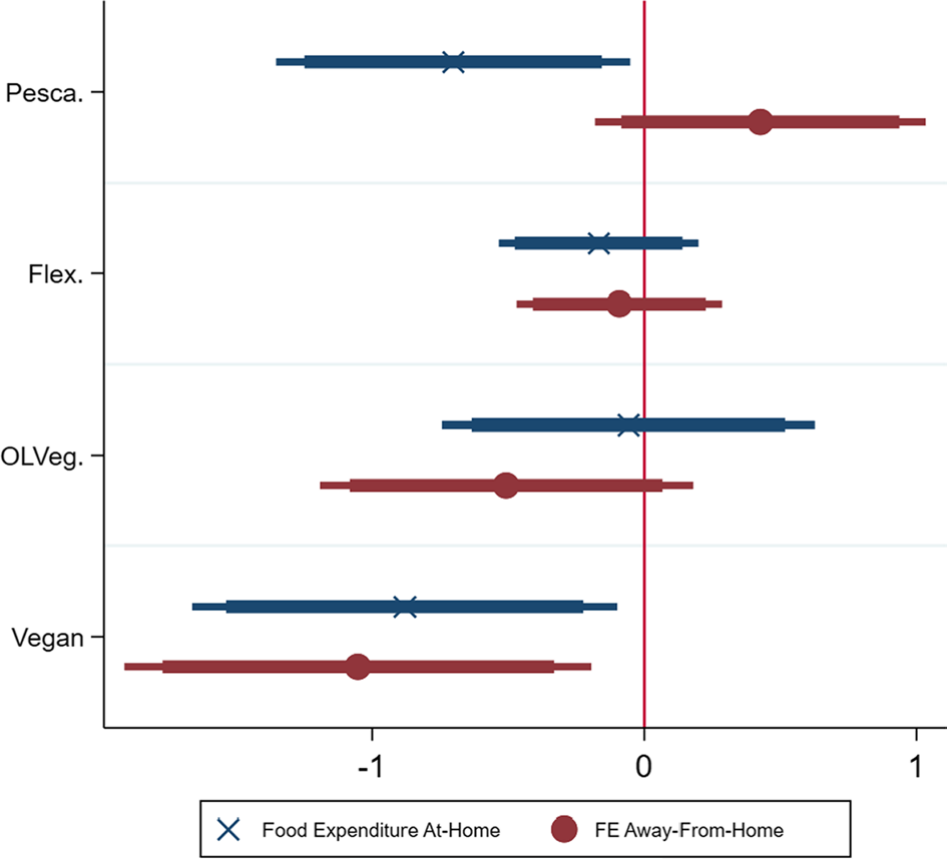
Coefficients of actual diets on food expenditure (Eqs. 2, 3)

**Fig. 4:**
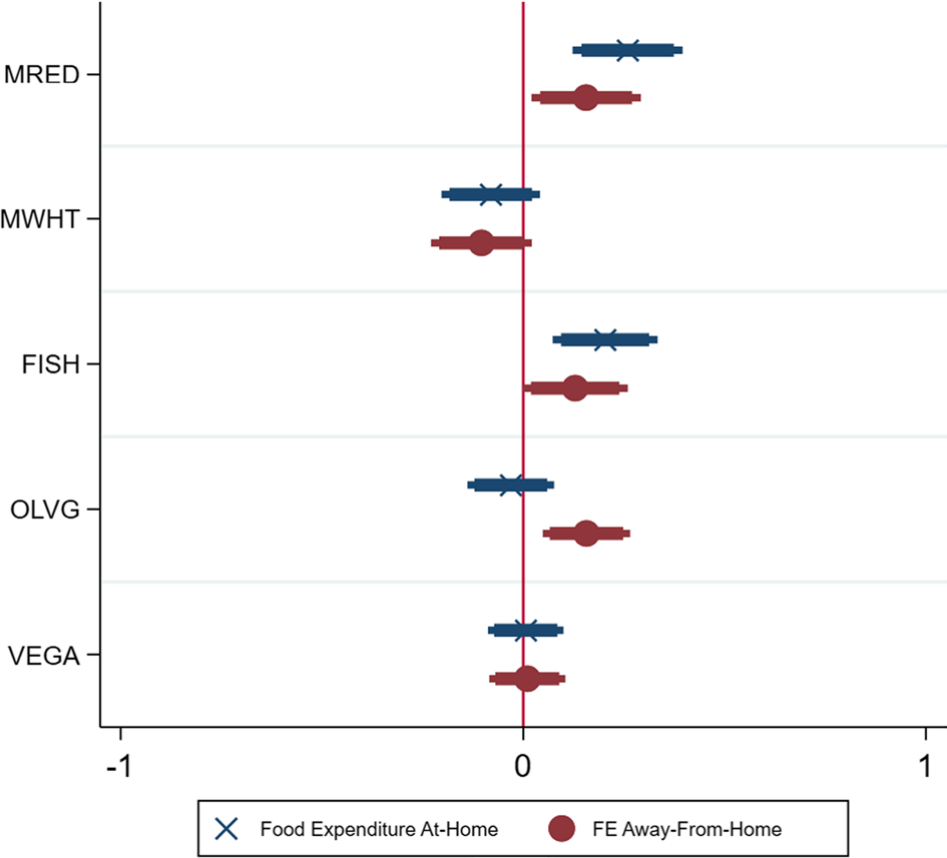
Coefficients of different meals on food expenditure (Eqs. 4, 5)

**Fig. 5:**
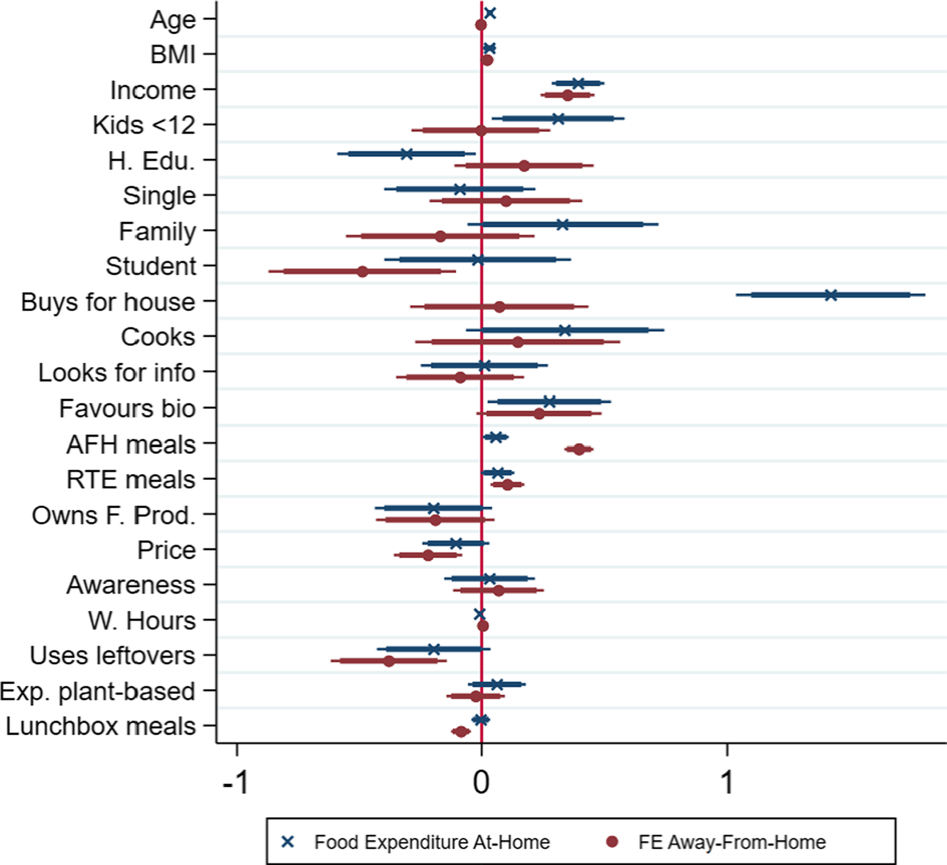
Coefficients of covariates for food expenditure (Eqs. 2, 3)

Following the hypotheses defined, Figs. 3, 4, and 5 show the coefficients^5^ of the models^6^ described in the general specifications (Eqs. 2–5). Although the coefficients should not be interpreted in terms of magnitude since they do not reflect a marginal change, the overall effect (positive or negative) can be derived. It is thus possible to test each hypothesis. Additionally, the marginal effects (Tables 5, 6, 7, 8) have the advantage to quantify the impact of an increase in the predictors’ value on each category of food expenditures, i.e., the impact of a one unit-increase on the likelihood of spending less than 20€, or on the likelihood of spending 60€–79€. These are analysed in Sect. 3.3. The econometric software STATA 15 was used.

Testing *H1: Plant-based diets, compared to omnivorous diets, positively affect the likelihood of spending more on food*, Fig. 3 shows that a vegan consumer, compared to an omnivorous one, is associated with a lower food expenditure, for both at-home (Eq. 1) and away-from-home (Eq. 2). This means that vegan consumers are less likely to spend more on food (or more likely to spend lower amounts of money, according to the detailed marginal effects described in Table 5) compared to omnivorous consumers. The same can be said about pescatarians for food expenditures at-home. Therefore, *H1a* (Eq. 2, $\beta_{115} > 0,$ *p* value = 0.014) and *H1b* (Eq. 3, $\beta_{115} > 0,$ *p* value = 0.008) are rejected and it can be concluded that plant-based consumers do not spend more, but rather less than omnivorous consumers, for the sample assessed. However, additional insights can be derived concerning the cost associated with the number of meals consumed; these are presented further.

Considering *H2: higher frequency of plant-based meals is associated with higher food expenditures*, Fig. 4 shows that there is no relationship between the number of vegan meals and food expenditures, for both at-home (Eq. 3) and away-from-home (Eq. 4). However, an increase in ovo-lacto-vegetarian meals is associated with higher food expenditures away-from-home. Additionally, an increase in red meat meals and fish meals is also associated with higher food expenditures, for both *FEAH* and *FEAFH*. Therefore, *H2a* (Eq. 4, $\beta_{5} > 0,$ *p* value = 0.55) and *H2b* (Eq. 5, $\beta_{5} > 0,$ *p* value = 0.582) cannot be rejected for plant-based diets (vegan). Nonetheless, eating more red meat and fish meals increases both food expenditures.

Following Fig. 5, the hypotheses concerning with food-related preferences can be tested^7^. First, *H3: informed consumers frequently spend less on food* can be verified through the variable *INFO* (looks for information before buying). The hypothesis cannot be rejected since the coefficient shows no statistical significance level either regarding at-home or away-from-home. Second, *consumers who favour biologic/organic foods tend to spend more on food* (*H4*) is not rejected as indeed results show a positive relationship between favour biologic and spending more on food both at-home (*H4a*) and away-from-home (*H4b*). Third, for *H5: consumers who cook for themselves and others, use leftovers, and own/receive local food production frequently spend less on food*, results show that only one aspect (use of leftovers) of a pro-active consumer is generally associated with lower food expenditures, while the other aspects do not show any statistically significant relationship.

Moreover, other inferences can be derived from Fig. 5. For example, consumers with a higher education spend less on food at-home, while the wealthier the consumer, the higher is the food expenditure both at-home and away-from-home. Consumers who eat ready-to-eat meals more frequently generally end up spending more, although ready-to-eat meals are generally perceived as cheaper and more convenient than preparing meals with fresh food. Furthermore, the expected effects from the rest of the covariates corroborate the good consistency of the models. For example, a consumer who buys for the household spends more than a consumer who buys only for herself/himself. The same is shown for a consumer who lives with her/his family, a consumer who spends more than another consumer who lives alone or shares the house with non-family members. Additionally, the number of away-from-home meals is positively associated with spending more away-from-home, while lunchbox meals show a negative effect. These effects are in accordance with what is expected from reality and thus reinforce the robustness of the models.

### Marginal effects and food policy implications

The marginal effects computed from the coefficients described above make it possible to assess the effect of the different diets on food expenditure in more detail, particularly for each category of spending. Tables 5, 6, 7 and 8 present the marginal effects associated with the different diets assessed for food expenditure at-home (*FEAH*). The * denotes statistical significance at the 5% level. As described above, both pescatarian and vegan consumers tend to generally spend less on food expenditure compared with omnivorous consumers. Table 5 suggests that, on average, following a vegan diet increases a consumer’s probability of spending 20–39€ and 40–59€ by 0.082 and 0.107, respectively, compared with an omnivorous diet. Additionally, the predicted probabilities of spending 120–139€, and 140€ or more is on average 0.034 and 0.048 lower for a vegan consumer than for an otherwise similar omnivorous consumer. Therefore, vegan consumers are more likely to spend lower amounts of money and less likely to spend higher amounts on food at-home compared with omnivorous consumers, which again rejects *H1a*, that plant-based consumers spend more than omnivorous consumers (at-home).

**Table 5:** Marginal effects of diets on food expenditure at-home

*FEAH*	< 20€	20–39€	40–59€	60–79€	80–99€	100–119€	120–139€	> 140€
P versus O	0.014	0.062*	0.089**	0.005	− 0.043*	− 0.057**	− 0.029**	− 0.041***
F versus O	0.003	0.012	0.022	0.005	− 0.008	− 0.014	− 0.008	− 0.012
OLV versus O	0.001	0.004	0.008	0.002	− 0.002	− 0.005	− 0.003	− 0.005
V versus O	0.02	0.082*	0.107***	− 0.001	− 0.057*	− 0.069**	− 0.034***	− 0.048***
F versus P	− 0.012	− 0.05	− 0.067	0	0.035	0.042	0.021	0.029
OLV versus P	− 0.013	− 0.058	− 0.081	− 0.003	0.041	0.052	0.026	0.037
V versus P	0.005	0.021	0.018	− 0.006	− 0.014	− 0.012	− 0.005	− 0.007
OLV versus F	− 0.002	− 0.008	− 0.014	− 0.003	0.005	0.009	0.005	0.008
V versus F	0.017	0.07	0.085**	− 0.006	− 0.049	− 0.055*	− 0.026*	− 0.036**
V versus OLV	0.019	0.078	0.099*	− 0.003	− 0.054*	− 0.064*	− 0.031*	− 0.044

Statistical significance levels of 10, 5, and 1% are denoted as *, **, and ***, respectively

Due to space constraints, the marginal effects of the covariates are available from the authors upon request

**Table 6:** Marginal effects of diets on food expenditure away-from-home

*FEAFH*	< 5€	5–9€	10–19€	20–39€	40–59€	60–79€	80–99€	> 100€
P versus O	− 0.055	− 0.038	− 0.008	0.047	0.029	0.014	0.006	0.006
F versus O	0.014	0.008	− 0.001	− 0.011	− 0.005	− 0.002	− 0.001	− 0.001
OLV versus O	0.088	0.036**	− 0.019	− 0.059	− 0.026*	− 0.011*	− 0.005*	− 0.005*
V versus O	0.208*	0.049***	− 0.065	− 0.113***	− 0.045***	− 0.018***	− 0.008***	− 0.008***
F versus P	0.07	0.046	0.007	− 0.058	− 0.035	− 0.016	− 0.007	− 0.007
OLV versus P	0.144**	0.074**	− 0.011	− 0.106**	− 0.055**	− 0.024*	− 0.011*	− 0.011*
V versus P	0.264**	0.087***	− 0.057	− 0.16***	− 0.074***	− 0.032**	− 0.014**	− 0.014**
OLV versus F	0.074	0.028	− 0.018	− 0.048	− 0.021	− 0.009	− 0.004	− 0.004
V versus F	0.194*	0.041***	− 0.064	− 0.102**	− 0.039**	− 0.016**	− 0.007**	− 0.007**
V versus OLV	0.12	0.013	− 0.046	− 0.054	− 0.019	− 0.007	− 0.003	− 0.003

Statistical significance levels of 10, 5, and 1% are denoted as *, **, and ***, respectively

Due to space constraints, the marginal effects of the covariates are available from the authors upon request

**Table 7:** Marginal effects of meals on food expenditure at-home

*FEAH*	< 20€	20–39€	40–59€	60–79€	80–99€	100–119€	120–139€	> 140€
MRED								
1+	− 0.003***	− 0.016***	− 0.033***	− 0.012***	0.008***	0.022***	0.013***	0.021***
MWHT								
1+	0.001	0.006	0.011	0.003	− 0.004	− 0.007	− 0.004	− 0.006
FISH								
1+	− 0.003***	− 0.013***	− 0.026***	− 0.009**	0.007***	0.017***	0.01***	0.016***
OLVG								
1+	0	0.002	0.004	0.001	− 0.001	− 0.003	− 0.001	− 0.002
VEGA								
1+	0	0	− 0.001	0	0	0.001	0	0

Statistical significance levels of 5, and 1% are denoted as **, and ***, respectively

1+  denotes one-unit increase. Due to space constraints, the marginal effects of the covariates are available from the authors upon request

**Table 8:** Marginal effects of meals on food expenditure away-from-home

*FEAFH*	< 5€	5–9€	10–19€	20–39€	40–59€	60–79€	80–99€	> 100€
MRED								
1+	− 0.023**	− 0.014**	0	0.018**	0.01**	0.004**	0.002*	0.002*
MWHT								
1+	0.016	0.009*	− 0.002	− 0.012	− 0.006*	− 0.003*	− 0.001	− 0.001
FISH								
1+	− 0.019**	− 0.011*	0	0.015*	0.008*	0.003*	0.002*	0.002*
OLVG								
1+	− 0.023***	− 0.014***	0	0.018***	0.01***	0.004**	0.002**	0.002**
VEGA								
1+	− 0.002	− 0.001	0	0.001	0.001	0	0	0

Statistical significance levels of 10, 5, and 1% are denoted as *, **, and ***, respectively

1+ denotes one-unit increase. Due to space constraints, the marginal effects of the covariates are available from the authors upon request

Regarding pescatarian consumers, similar marginal effects are found, although at lower predicted probabilities. The coefficients described in Fig. 3 only show the effect of the different diets analysed comparing them to an omnivorous diet as the base. Table 5, however, shows all the possible associations between the five diets assessed. These results show that a vegan consumer is more likely to spend lower amounts of money on food and less likely to spend higher amounts than a flexitarian. Hence, it is possible to conclude that vegans also tend to spend less on food at-home than otherwise similar flexitarians. Nevertheless, the marginal effects of vegans are higher when compared with omnivorous consumers, followed by ovo-lacto-vegetarians and finally flexitarians.

When analysing food expenditure away-from-home, the results described in Table 6 show similar patterns for plant-based and omnivorous diets. Vegan consumers are more likely to spend amounts less than 10€ and less likely to spend any other amount above 9€, compared with omnivorous consumers, for a statistical significance level of 1%. No statistically significant difference in food expenditure away-from-home was found between pescatarians and omnivorous consumers. Ovo-lacto-vegetarians, however, show higher probabilities of spending lesser amounts, compared with omnivorous consumers (although only at a 10% significance level). Additionally, plant-based consumers also tend to spend less on food away-from-home when compared with both pescatarians and flexitarians (which is also true for vegetarians compared with pescatarians). Therefore, to further corroborate previous findings, vegan consumers are more likely to spend lower amounts and less likely to spend higher amounts of money on food away-from-home compared with all other diets except ovo-lacto-vegetarians, rejecting *H1b* that plant-based consumers spend more than omnivorous consumers (away-from-home).

The marginal effects concerning food choices presented in Table 7 are interpreted differently, since the variables are ordinal instead of nominal. On average, a one unit-increase in the frequency of red meat meals is associated with a 0.021 increase in the probability of spending 140€ or more, and a 0.033 decrease in the probability of spending 40–59€ on food at-home. These marginal effects have a statistical significance level of 1%. Fish meals show similar marginal effects, although with lower magnitudes. Therefore, results show that an increase in red meat meals and fish meals per week increases the amount spent on food consumed at-home.

Since omnivorous diets include red meat and fish at considerable levels, at least with no intention of reduction (which are the flexitarian diets), results suggest that omnivorous consumers increase their food expenditure if the frequency of meals increases. The same goes for pescatarians and fish meals, while the frequency of vegetarian and vegan meals is not statistically significant. As consumers cannot eat infinitely, they incur trade-offs and choose between different meals. Choosing red meat meals, for example, incurs an opportunity cost of not choosing white meat meals or vegan meals, which could be cheaper than red meat. This explains why the positive association was found for red meals and fish meals.

Concerning food expenditure away-from-home, red meat meals show similar marginal effects as for food expenditure at-home (Table 8). The marginal effect of red meat meals is higher for the probability of spending less than 5€, i.e., a one unit-increase in the frequency of consuming a red meat meal is associated with a 0.023 decrease in the probability of spending less than 5€. The marginal effects found for fish meals are statistically significant only at the 10% level, while ovo-lacto-vegetarian meals show a similar marginal effect as red meat meals. A one unit-increase in the frequency of vegetarian meals is associated with a decrease in the probability of spending lower amounts on food away-from-home (less than 5€, and 5–9€), while spending higher amounts increases.

Globally, the results suggest that Portuguese consumers who follow a plant-based diet end up spending less on both food consumed at-home and away-from-home, compared with an otherwise similar Portuguese consumer who follows an omnivorous diet. The same can be said when compared with flexitarians. Evidence also shows that plant-based consumers spend less than pescatarians away-from-home. These insights, particularly that plant-based consumers spend less than omnivorous consumers on a weekly basis, follow the findings highlighted in the literature for the USA (Lusk and Norwood 2016), Sweden (Grabs 2015), and the UK (Berners-Lee et al. 2012; Hoolohan et al. 2013). To the authors’ knowledge, the present study is the first to assess these expenditure patterns in a Mediterranean country with strong cultural dietary roots, such as Portugal.

To promote healthy and sustainable diets, these also need to be affordable for all. Considering that plant-based diets are often highlighted in the literature as healthier and more sustainable, according to the present study, plant-based consumers are also shown to spend less than consumers following other diets. Thus, promoting plant-based diets as possibly cheaper can also be advantageous for achieving more acceptance and reaching a wider range of consumers, as price is an important factor when purchasing, particularly for poorer consumers. This away, policymakers can use plant-based diets as a mechanism to mitigate, not only climate change, but also food insecurity present in poorer households. Additionally, since plant-based consumers spend less on food, there will be a saving which will be spent elsewhere. Thus, a plant-based consumer will have a greater budgetary availability for meeting other needs. However, from an environmental point-of-view, in some cases, these other needs could have a negative impact which could offset the environmental benefits of choosing a vegan diet. In these cases, the rebound effect of the savings should be considered to estimate the net effect.

## Conclusion and future research

Following the call from international organizations and the scientific community for a drastic change in dietary habits to ensure public health and the planet’s sustainability, plant-based diets have been promoted worldwide. However, considering that price is a major factor when purchasing food, it is vital that these healthier and more sustainable diets are also affordable. Making use of a representative sample of the population (*n* = 1040), the present study has tested, at the consumer level, if plant-based consumers spent more on food than omnivorous consumers.

Results suggest that plant-based consumers do not spend more but in fact less than any consumer assessed. This could be a promising feature for the promotion of plant-based diets, with particular interest for consumers with lower incomes by ensuring food security. According to the literature, plant-based diets can be healthier and more sustainable. Following the results of the present analysis, plant-based diets can also be cheaper. Additionally, increasing red meat meals is associated with an increase in food expenditures. This effect could be due to the opportunity cost of not choosing an alternative cheaper meal such as white meat or plant-based meals. The insights described from Portugal can be helpful for countries following the Mediterranean diet in the South of Europe. It would be worth exploring if similar patterns are found in other countries.

Some limitations and future improvements in the study should be noted. First, it is always unclear whether the omitted-variable bias exists because the “true” model is unknown. Thus, future research may include more covariates other than the ones considered here to minimize the bias. Moreover, studies like the present study rely on consumers’ capacity to honestly report information on the food consumed. Future research may consider other methodologies that can actually observe and report all foods consumed and the cost associated with them. This way, it will also be possible to capture other personal, cultural, socio-economic, and behavioural characteristics of the consumers which are difficult to assess using the present methodology. However, data of this nature would be expensive to collect.

Besides the active population (15–64 years), it is also important for future research to assess the food consumption of the population over 65 years old. This particular segment of the population introduces specific dietary habits to the analysis not found in other age groups, mainly due to health issues or simply aging issues. Also, this group generally eats less overall and “away-from-home”, thus being a relevant segment to assess. Additionally, it would also be interesting for future research to assess the consumers’ perception on the expenditure differences between animal- and plant-based diets. Do consumers perceive plant-based diets as more or less expensive? If consumers’ expectations are that plant-based diets are more expensive, and this perception acts as a barrier to change, then the results presented here are crucial to help consumers make informed choices.

## Data Availability

The data sets generated and analysed during the current study are not publicly available because they include data from an online survey which, if publicly available, may compromise the individual privacy of the respondents. Only the authors are authorized to use such protected data. However, the data could be available from the authors on reasonable request.

## Appendix

See Tables

**Table 9:** Items used for the construct *Food Awareness* (*AWAR*)

Q: In terms of information, how often have you heard about these subjects? (1 = never heard–5 = heard a lot)				
	Mean	Std. Dev.	Min	Max
Farm animal well-being abuse	4.174	0.964	1	5
Mad cow disease	4.241	0.858	1	5
Bird flu	4.285	0.815	1	5
Greenhouse gas emissions from livestock	4.206	1.026	1	5
Genetically modified food	4.257	0.872	1	5
Salmonella	4.210	0.909	1	5
Swine flu	4.104	0.918	1	5
Antibiotic use in livestock	3.935	1.180	1	5
Pesticides used in fruits and vegetables	4.417	0.768	1	5
Growth hormones use in livestock	4.139	1.039	1	5
E. coli	3.589	1.404	1	5
Pink Slime	1.656	1.082	1	5
Gestational stalls	2.129	1.271	1	5
Beak trimming	1.968	1.255	1	5
Cancer and meat consumption	3.995	1.064	1	5

The Cronbach’s alpha for the 15 items is 0.892, suggesting that the items have relatively high internal consistency

9 and

**Table 10:** Likelihood ratio test comparing probit versus logit models

Likelihood criteria	Probit	Logit	L–P
Equation 2—*FEAH*			
AIC	3821.365	3815.994	5.370
BIC (*df* = 31/31/0)	3979.668	3974.297	5.370
Result: difference of 5.370 in BIC provides ***positive*** support for logit model			
Equation 3—*FEAFH*			
AIC	3429.889	3422.091	7.798
BIC (*df* = 31/31/0)	3588.193	3580.395	7.798
Result: difference of 7.798 in BIC provides ***strong*** support for logit model			
Equation 4—*FEAH*			
AIC	3809.935	3802.539	7.396
BIC (*df* = 32/32/0)	3973.185	3965.790	7.396
Result: difference of 7.396 in BIC provides ***strong*** support for logit model			
Equation 5—*FEAFH*			
AIC	3423.081	3414.133	8.948
BIC (*df* = 32/32/0)	3586.331	3577.383	8.948
Result: difference of 8.948 in BIC provides ***strong*** support for logit model			

10.

## Footnotes

1 A copy of the survey is available from the authors upon request.

2 For additional information, including reports, raw data, and survey instrument files, consult Kansas State University’s website: https://www.agmanager.info/livestock-meat/meat-demand/monthly-meat-demand-monitor-survey-data.

3 Recorded as ordinal variables with five categories: (1) zero meals, (2) one to two meals, (3) three to four meals, (4) five to six meals, and (5) seven and more meals.

4 Information regarding both AIC and BIC criteria is presented in Table 10, in the Appendix.

5 The thick and thin lines denote 10 and 5% statistical significance levels, respectively. The variable is statistically significant when the lines do not cross the *x*-axis origin. The value on the *x*-axis is that of the coefficients.

6 Due to space constraints, the tables reporting the coefficients are available from the authors, upon request.

7 Since the coefficient effects of the covariates from Eqs. 2, 3 and 4, 5 are highly similar, only the ones from Eqs. 2, 3 are presented.
